# Notochordal-cell derived extracellular vesicles exert regenerative effects on canine and human nucleus pulposus cells

**DOI:** 10.18632/oncotarget.21483

**Published:** 2017-10-04

**Authors:** Frances Bach, Sten Libregts, Laura Creemers, Björn Meij, Keita Ito, Marca Wauben, Marianna Tryfonidou

**Affiliations:** ^1^ Department of Clinical Sciences of Companion Animals, Faculty of Veterinary Medicine, Utrecht University, Utrecht, The Netherlands; ^2^ Department of Biochemistry and Cell Biology, Faculty of Veterinary Medicine, Utrecht University, Utrecht, The Netherlands; ^3^ Department of Orthopaedics, University Medical Centre Utrecht, Utrecht, The Netherlands; ^4^ Orthopaedic Biomechanics, Department of Biomedical Engineering, Eindhoven University of Technology, Eindhoven, The Netherlands

**Keywords:** extracellular vesicles, intervertebral disc, notochordal cell, regeneration, nucleus pulposus

## Abstract

During intervertebral disc ageing, chondrocyte-like cells (CLCs) replace notochordal cells (NCs). NCs have been shown to induce regenerative effects in CLCs. Since vesicles released by NCs may be responsible for these effects, we characterized NC-derived extracellular vesicles (EVs) and determined their effect on CLCs.

EVs were purified from porcine NC-conditioned medium (NCCM) through size exclusion chromatography, ultracentrifugation or density gradient centrifugation. Additionally, the EVs were quantitatively analyzed by high-resolution flow cytometry. The effect of NCCM-derived EVs was studied on canine and human CLC micro-aggregates *in vitro* and compared with NCCM-derived proteins and unfractionated NCCM.

Porcine NCCM contained a considerable amount of EVs. NCCM-derived EVs induced GAG deposition in canine CLCs to a comparable level as NCCM-derived proteins and unfractionated NCCM, and increased the DNA and glycosaminoglycan (GAG) content of human micro-aggregates, although to a lesser extent than unfractionated NCCM. The biological EV effects were not considerably influenced by ultracentrifugation compared with size exclusion-based purification. Upon ultracentrifugation, interfering GAGs, but not collagens, were lost. Nonetheless, collagen type I or II supplemented to CLCs in a concentration as present in NCCM induced no anabolic effects.

Porcine NCCM-derived EVs exerted anabolic effects comparable to NCCM-derived proteins, while unfractionated NCCM was more potent in human CLCs. GAGs and collagens appeared not to mediate the regenerative EV effects. Thus, NC-derived EVs have regenerative potential, and their effects may be influenced by the proteins present in NCCM. The optimal combination of NC-secreted factors needs to be determined to fully exploit the regenerative potential of NC-based technology.

## INTRODUCTION

Low back pain, affecting up to 85% of the population and resulting in considerable socioeconomic consequences [1, 2], has been associated with intervertebral disc (IVD) degeneration [3]. Since dogs experience back pain and IVD degeneration with similar characteristics as humans, they are considered a suitable animal model for human IVD degeneration [4, 5]. The healthy IVD provides stability and flexibility to the spine and consists of a hydrated nucleus pulposus (NP) surrounded by the annulus fibrosus (AF). The NP, composed of glycosaminoglycan (GAG) and collagen type II, is derived from the notochord [6]. GAGs indirectly attract water, and in this way the IVD provides a shock absorption function for the spine. During maturation, notochordal cells (NCs) are replaced by chondrocyte-like cells (CLCs) in the NP. When the IVD degenerates, the CLCs are not able to maintain healthy tissue anymore. The CLCs become depleted, the GAG and water content decreases and collagen type II is replaced by collagen type I, resulting in a more fibrous tissue. The avascular IVD shows inadequate repair, and a vicious circle develops in which the IVD experiences increased vulnerability to damage by physiologic loading [7].

Current treatments for IVD disease aim at relieving symptoms, but do not address the underlying degeneration. Therefore, regenerative strategies have gained increased attention [8–10]. Successful treatment strategies can be developed by mimicking developmental biology. In this respect, NCs have attracted increasing interest because of their potential regenerative capacity [11]. Large, vacuolated NCs are only present in the NP of young human individuals and disappear around 10 years of age. The replacement of NCs by CLCs precedes the onset of IVD degeneration, implying that NCs may play a role in maintaining IVD health. The regenerative effect of NC-conditioned medium (NCCM) has already been demonstrated on CLCs [12–14], mesenchymal stromal cells (MSCs) [15–17], and NP tissue explants [18] *in vitro*, and on rat IVDs *in vivo* [19]. NCCM may exert its effects in several ways: through extracellular matrix (ECM) components such as GAGs [20] and/or through growth factors. Factors that were already found are connective tissue growth factor (CTGF) [19, 21], transforming growth factor-β_1_ (TGF-β_1_), Wnt-induced soluble protein 2, insulin-like growth factor binding protein 7, and angiopoietin-like 7 [19] in canine NCCM, and CTGF [22], alpha-2-macroglobulin, clusterin, and tenascin [16] in porcine NCCM.

Recently, extracellular vesicles (EVs) have gained increased attention. EVs are small, membrane-enclosed particles released by cells that play a role in intercellular signaling [23], and are involved in tissue regeneration [24, 25]. We previously proposed that EVs may be responsible for regenerative NCCM effects [26]. The anabolic effect of pelletable (theoretically containing EVs and protein aggregates) NCCM factors was, however, less pronounced than that of soluble (peptides, proteins) NCCM factors [26]. This observation could be attributed to the ultracentrifugation (UC) procedure that may negatively affect the biological EV properties [27] or to the interfering protein aggregates present in the pelletable fraction [28]. Therefore, the first aim of the current study was to purify and characterize NC-derived EVs from porcine NCCM. The second aim was to determine the biologic effect of the NCCM-derived EVs on canine and human CLCs from degenerated IVDs *in vitro*.

## RESULTS

### NCCM-derived extracellular vesicle purification and characterization

NCCM-derived EVs were separated from soluble proteins by size-exclusion chromatography (SEC) and separately pelleted by UC at 100,000*g*. The UC pellets (containing either EVs or proteins) were labelled with PKH67. Thereafter, the EVs were floated in sucrose gradients to their buoyant density. Lastly, the EVs were quantitatively analyzed by high-resolution flow cytometry.

To confirm the presence of true EV within samples upon flow cytometric analysis, detergents can be added to disrupt the EV lipid bilayer [29]. Upon addition of 0.1% SDS or 0.1% Triton X-100 to the samples containing the highest number of EVs (density 1.12 g/mL), the event rate reduced considerably (Supplementary Figure 1a). This was accompanied by a loss of light scattering events (Supplementary Figure 1a). Taken together, this confirms that the majority of detected events during flow cytometric analysis were indeed EVs. To demonstrate that during flow cytometric analysis single EVs were measured and not EV swarms (presence of multiple EVs within the measuring spot [30]), the 1.12 g/mL sucrose gradient fractions were serially diluted and analyzed. Upon dilution, the scatter profiles of EVs did not change (Supplementary Figure 1c). A linear correlation (R^2^: 0.9996) was found between the number of measured events and the dilution (Supplementary Figure 1b), indicating that single EVs and not swarms of EVs were detected.

High-resolution flow cytometry of porcine NCCM-derived EV and protein (P) 100,000*g* UC pellets revealed that the number of EVs differed considerably between donors (Supplementary Figure 2). The majority of EVs were, however, detected at the same densities. Additionally, the EV scatter profiles were comparable between all donors (Figure 2). In all donors, the highest number of events was measured in the 1.10, 1.12, and 1.14 g/mL sucrose gradient fractions of the EV 100,000*g* UC pellets. In the P 100,000*g* UC pellets, most events were also detected in the 1.10-1.14 g/mL sucrose gradient fractions, but the number of events was significantly lower compared to the EV 100,000*g* UC pellets (*p*<0.05).

**Figure 1:**
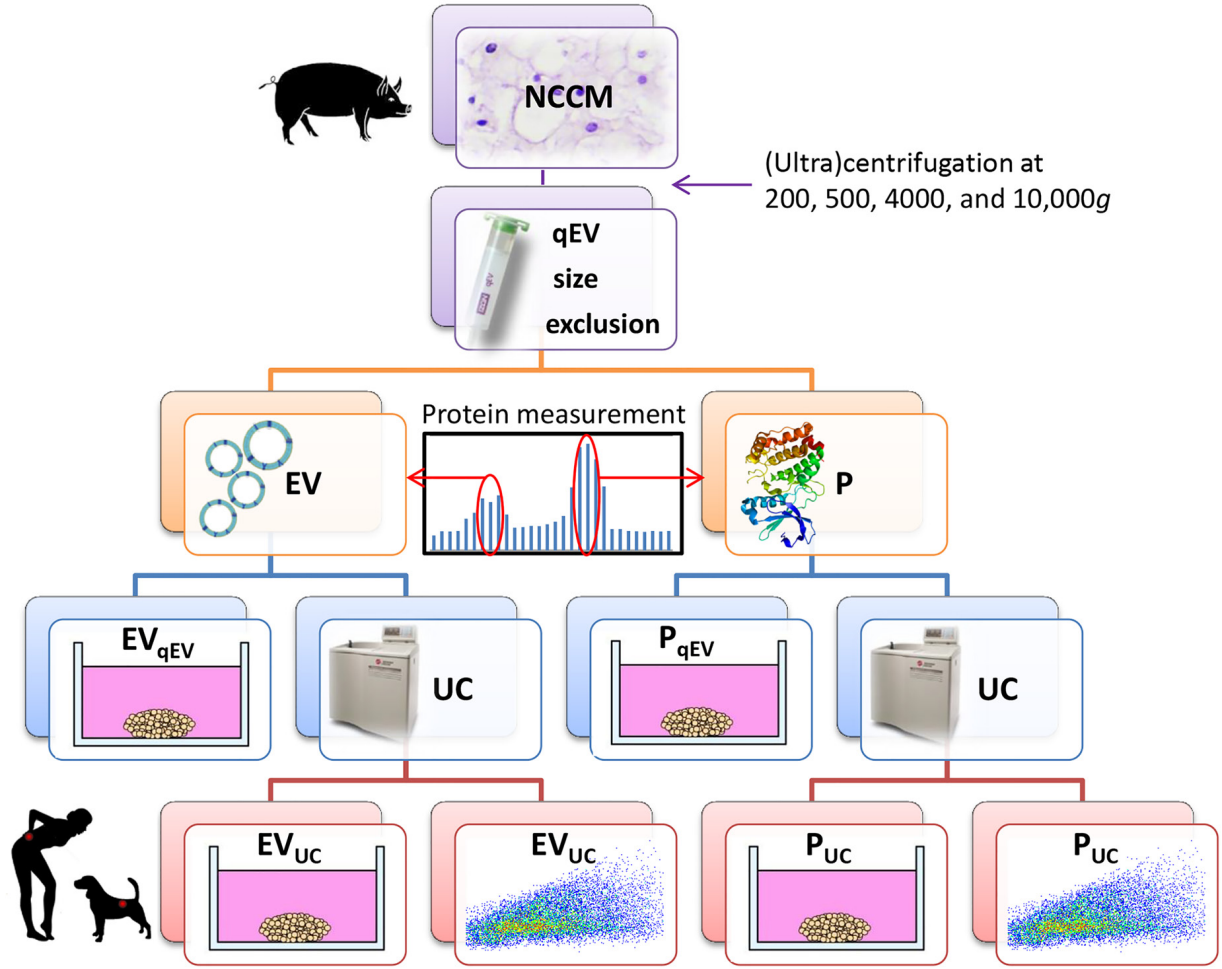
Schematic representation of the experimental setup Notochordal cell-conditioned medium (NCCM) was generated by culturing porcine NC-rich nucleus pulposus tissue for 4 days and (ultra) centrifuged to remove cells and cell debris. For extracellular vesicle (EV) purification, 10,000*g* NCCM supernatant was subjected to size-exclusion chromatography (SEC). Based on expected EV size and protein measurements, the three fractions with most EVs and proteins (P) were separately collected and pooled (4.5 mL). Part (1.5 mL) was directly used in micro-aggregate culture experiments (EV_qEV_ and P_qEV_), and part (3 mL) was subjected to ultracentrifugation (UC) at 100,000*g* for EV enrichment. The 100,000*g* UC pellet (40 μL) was then partly (35 μL) used in culture experiments (EV_UC_ and P_UC_), whereas the remainder (5 μL) was used for quantitative EV analysis using high-resolution flow cytometric analysis. *n* = 7 porcine NCCM donors, tested on a pool of 4 canine (*in triplo*) and 4 human chondrocyte-like cell donors (*in duplo*) in culture.

**Figure 2:**
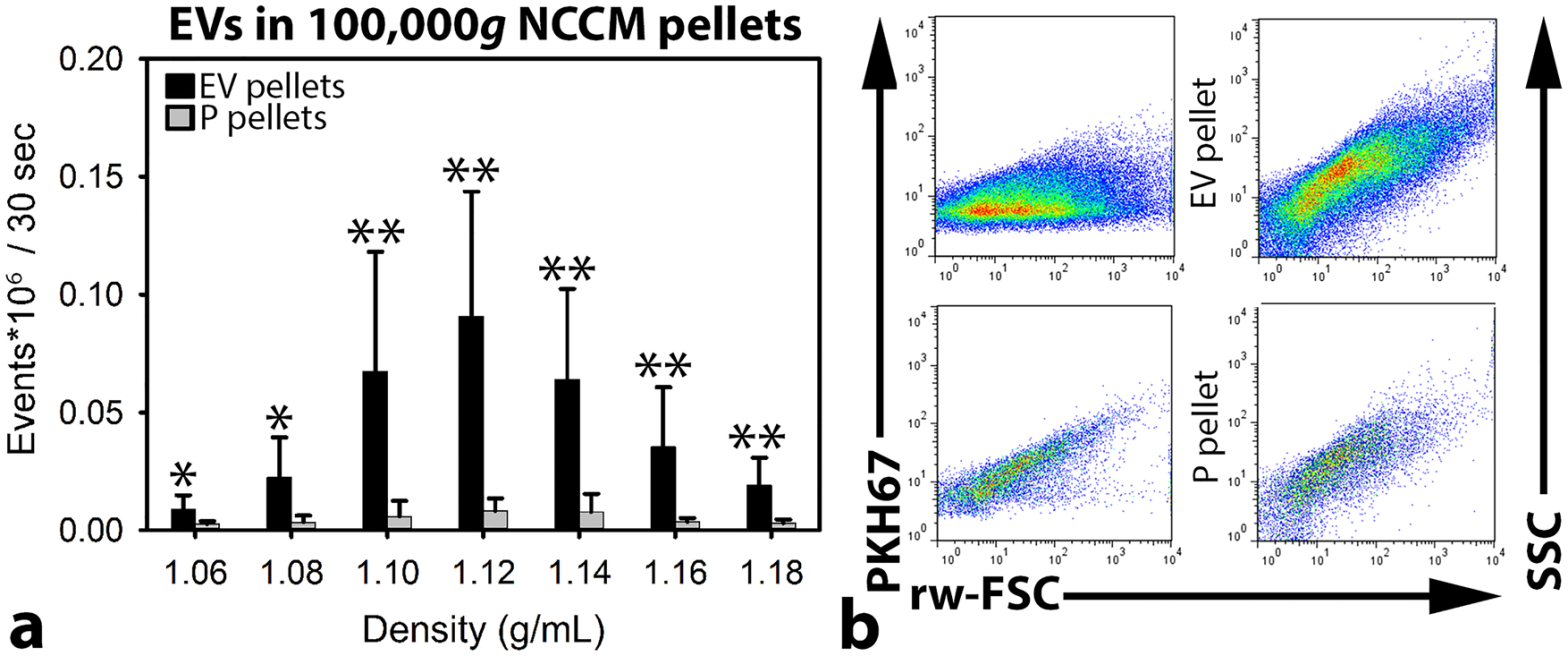
Quantitative extracellular vesicle (EV) analysis by high-resolution flow cytometry of notochordal cell-conditioned medium (NCCM) **(a)** Number of measured events per sucrose gradient fraction (with different densities) (+ SD) from floated porcine NCCM EV 100,000*g* ultracentrifugation (UC) pellets (black bars) and floated protein (P) 100,000*g* UC pellets (grey bars). *,**: significantly more measured events per 30 sec in the sucrose gradient fractions of the EV 100,000*g* UC pellets than in those of the P 100,000*g* UC pellets (*p*<0.05, *p*<0.01, respectively). **(b)** Dot plots of the sucrose gradient fraction with the highest number of measured events (density 1.12 g/mL) from one representative donor (donor 2). Dot plots represent levels of PKH67 intensity (left) or side scatter (SCC; right) (y-axis) versus reduced wide-angle forward scatter (rw-FSC) (x-axis). Upper dot plots represent the 1.12 g/mL sucrose fraction of the EV 100,000*g* UC pellet, and lower dot plots represent the 1.12 g/mL sucrose fraction of the P 100,000*g* UC pellet. *n* = 7 porcine NCCM donors.

### The effect of porcine NCCM-derived purified extracellular vesicles and proteins on canine and human CLCs

#### NCCM-derived EVs and proteins induce GAG deposition in canine and human CLC micro-aggregates

**Figure 3:**
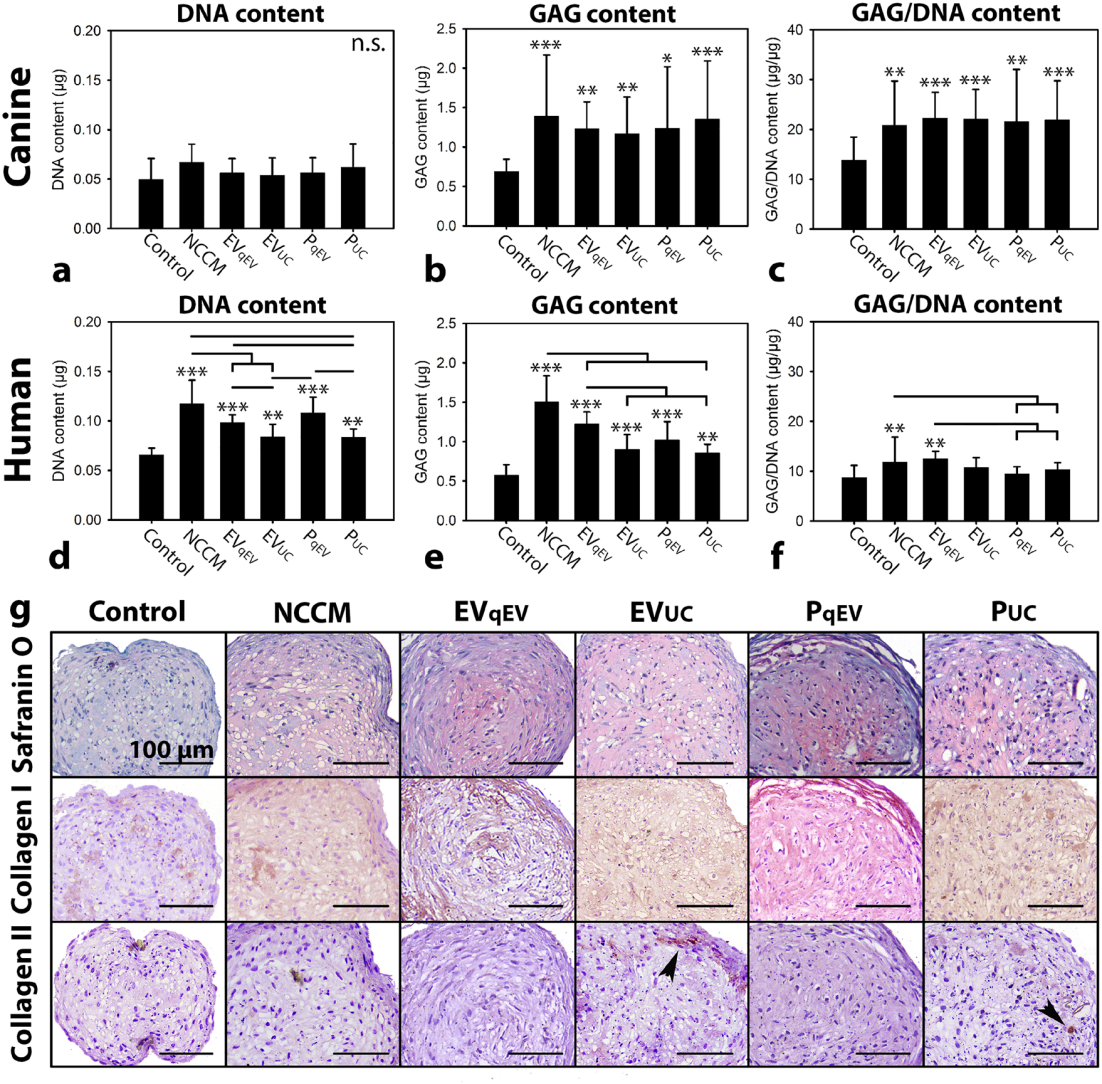
Notochordal cell-conditioned medium (NCCM)-derived extracellular vesicles (EVs) and proteins (P) induce increased glycosaminoglycan (GAG) deposition in chondrocyte-like cell (CLC) micro-aggregates derived from degenerated discs **(a, d)** DNA content **(b, e)** GAG content, **(c, f)** GAG/DNA content (mean ± SD) of canine and human CLC micro-aggregates, respectively, cultured in control culture medium, unfractionated porcine NCCM, or porcine NCCM-derived EVs or proteins for 7 (canine) or 21 (human) days. EV_qEV_: NCCM-derived EVs obtained after size exclusion chromatography (SEC), EV_UC_: NCCM-derived EVs obtained after SEC and subsequent 100,000*g* ultracentrifugation (UC), P_qEV_: NCCM-derived proteins obtained after SEC, P_UC_: NCCM-derived proteins obtained after SEC and subsequent 100,000*g* UC. Bars indicate significant differences between conditions (*p*<0.05); *, **,***: significantly different from controls (*p*<0.05, *p*<0.01, *p*<0.001, respectively); **(g)** Safranin O/Fast Green staining and collagen type I and II immunohistochemistry of human CLC micro-aggregates after 21 days of culture. Arrowheads indicate collagen type II deposition. *n* = 7 porcine NCCM donors tested on a pool of 4 canine (*in triplo*) and 4 human CLC donors (*in duplo*). n.s.: not significantly different.

Based on the expected EV sizes and protein measurements, the three NCCM SEC fractions with most EVs and proteins (P) were separately collected (Supplementary Figure 3). Part was directly used in culture (EV_qEV_ and P_qEV_), and part was subjected to 100,000*g* UC and thereafter used in culture (EV_UC_ and P_UC_). After 7 days of culture, no treatment significantly influenced the canine micro-aggregates’ DNA content compared with controls (Figure 3a). The GAG and GAG/DNA content of the canine micro-aggregates were, however, significantly increased by 7-day unfractionated NCCM, EV_qEV_, EV_UC_, P_qEV_, and P_UC_ treatment (*p*<0.05), with no differences between these treatments (Figure 3b and 3c).

The DNA and GAG content of the human micro-aggregates were significantly induced by 21-day unfractionated NCCM, EV_qEV_, EV_UC_, P_qEV_, and P_UC_ treatment (*p*<0.01, Figure 3d, 3e, and 3g). Unfractionated NCCM was most potent in this respect, followed by EV_qEV_ and P_qEV_, and lastly EV_UC_ and P_UC_. EV_qEV_ induced a similar DNA and GAG content in human micro-aggregates as P_qEV_ treatment. Also EV_UC_ was equally potent in increasing the DNA and GAG content of the human micro-aggregates as P_UC._ Both were, however, less potent than EV_qEV_ and P_qEV_ in this respect. Only 21-day EV_qEV_ and unfractionated NCCM treatment significantly increased the GAG/DNA content of the human micro-aggregates compared with control, P_qEV_ and P_UC_ treatment (*p*<0.05, Figure 3f). All treatments increased collagen type I deposition compared with controls (Figure 3g). Only in micro-aggregates treated with EV_UC_ and P_UC_, limited collagen type II was deposited (Figure 3g).

The total number of EVs in the sucrose fractions (density 1.06-1.18 g/mL) of the porcine NCCM EV 100,000*g* UC pellets did not correlate with the GAG content of the canine and human micro-aggregates treated with these EVs (Supplementary Figure 4a-4h). Notably, the number of measured EVs and the canine, but not the human, micro-aggregates’ GAG/DNA content after 7-day EV_qEV_ treatment displayed a moderate correlation (*r*:0.600, *p*<0.05; Supplementary Figure 4c).

#### Collagen and GAG concentration of the different culture media

**Figure 4:**
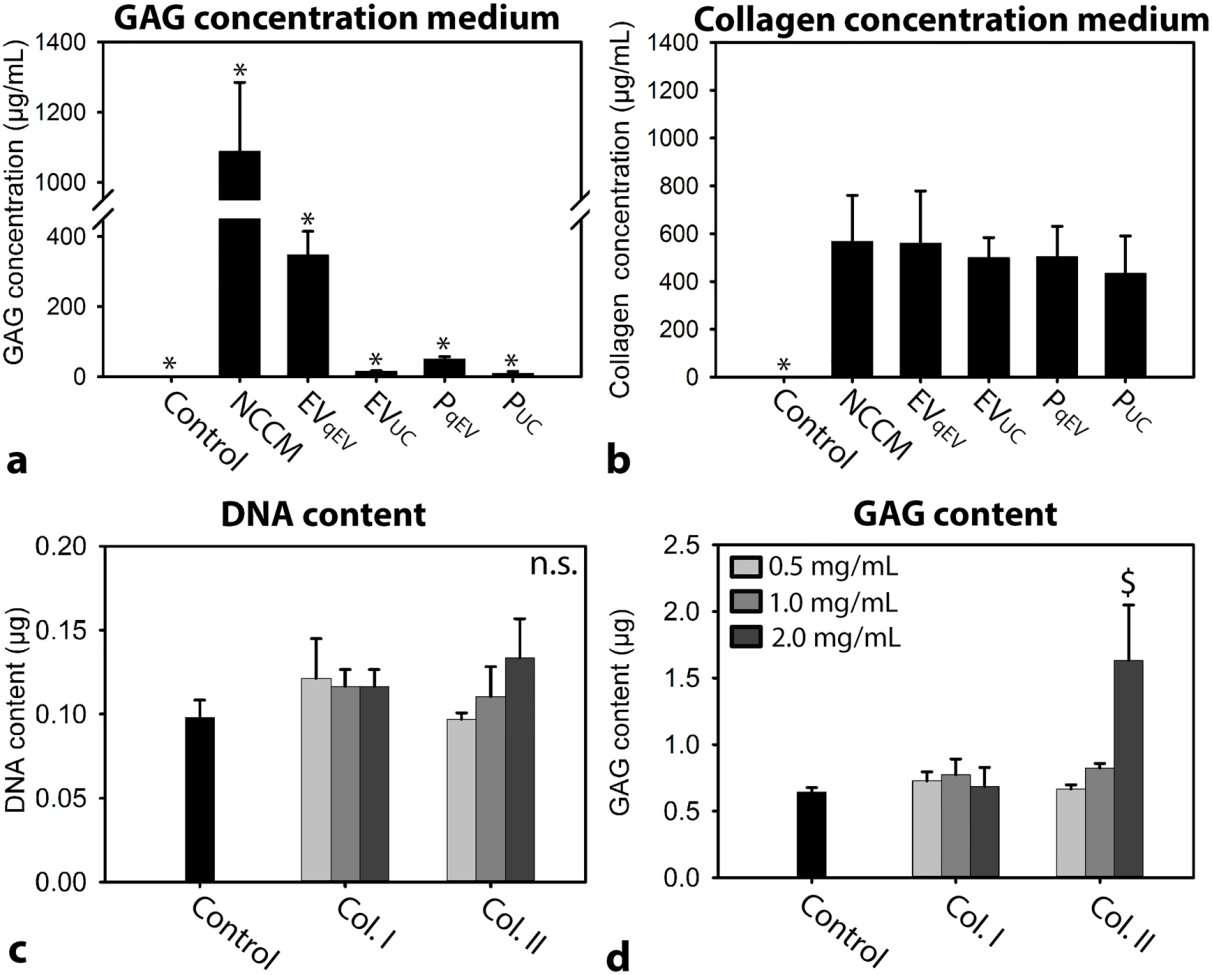
**(a)** GAG and **(b)** collagen concentration of the different culture media. *n* = 7 porcine NCCM donors. **(c)** DNA content **(d)** GAG content (mean + SD) of canine CLC micro-aggregates cultured in basal culture medium (control), supplemented with/without 0.5, 1.0 or 2.0 mg/mL collagen type I or II for 7 days. *n* = 6, tested on a pool of 4 canine CLC donors. *: significantly different from all other conditions (*p*<0.05); $: significantly different from control (*p*<0.05); GAG: glycosaminoglycan. n.s.: not significantly different.

The GAG concentration of unfractionated NCCM, EV_qEV_, EV_UC_, P_qEV_, and P_UC_ media was significantly higher than that of control media (in which GAGs were undetectable; *p*<0.05; Figure 4a). The GAG concentration of unfractionated NCCM was the highest, followed by EV_qEV_, P_qEV,_ EV_UC,_ and lastly P_UC_ (*p*<0.05). This indicates that with 100,000*g* UC, GAGs were lost. The collagen concentration of unfractionated NCCM, EV_qEV_, EV_UC_, P_qEV_, and P_UC_ media was significantly higher than that of control media (in which collagen was undetectable; *p*<0.05; Figure 4b). No differences in collagen concentration were detected between NCCM, EV, and P media.

Since in all NCCM-derived culture media collagen was present, it was determined whether collagen alone could exert effects on CLCs when it was applied at a concentration as present in NCCM(-derived EV/P media) (0.5 mg/mL). Interestingly, 0.5 and 1 mg/mL collagen type I or II supplementation did not exert regenerative effects on canine CLCs (Figure 4c and 4d). Only 2.0 mg/mL collagen type II increased the canine micro-aggregates GAG content compared with controls (*p*<0.05).

#### Serial dilution of NCCM(-derived EVs and proteins)

**Figure 5:**
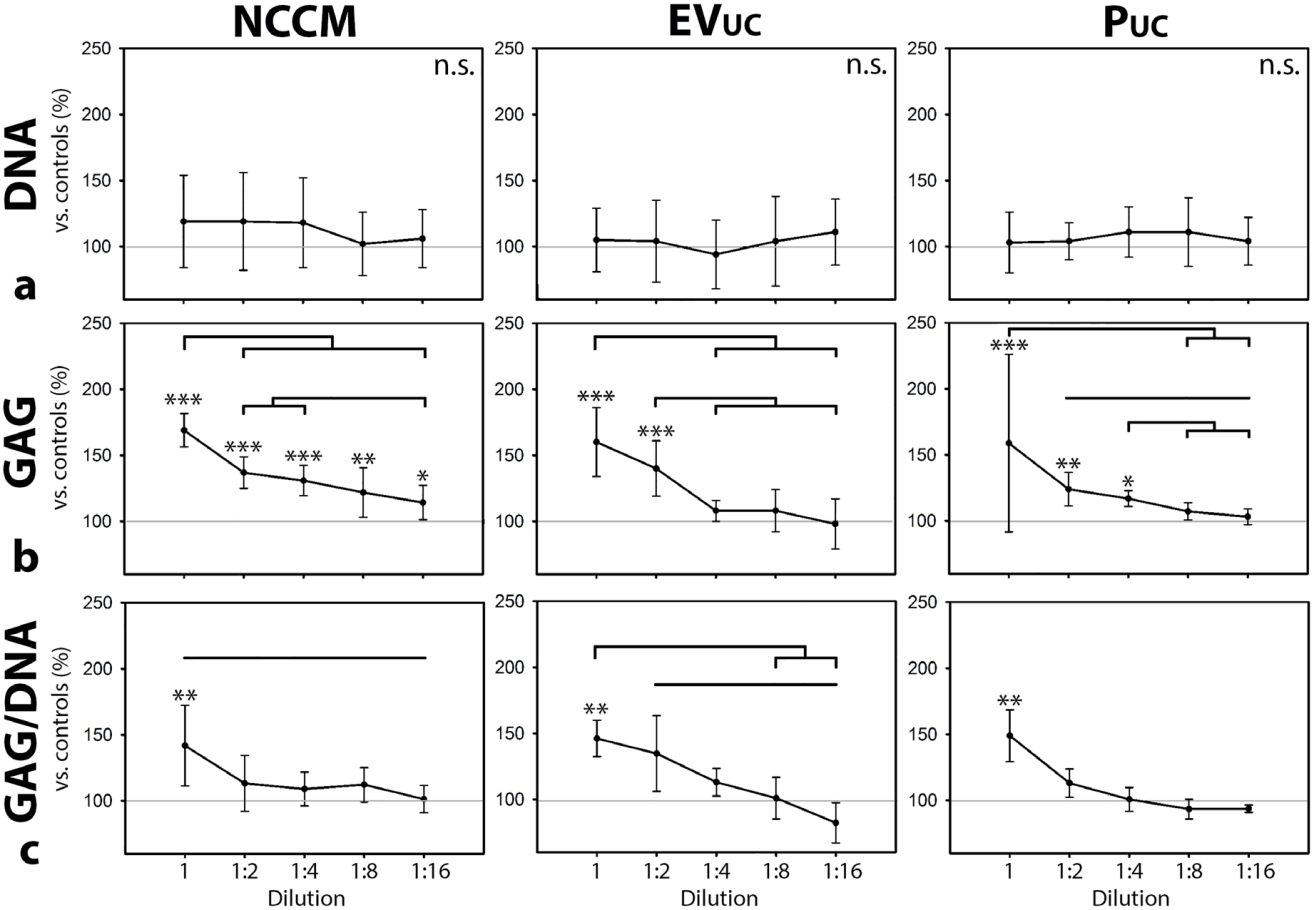
Serial dilution of notochordal cell-conditioned medium (NCCM), and NCCM-derived extracellular vesicles (EVs) and proteins (P) **(a)** DNA content, **(b)** GAG content, (**c**) GAG/DNA content (mean ± SD) of canine CLC micro-aggregates cultured for 7 days in control culture medium, unfractionated porcine NCCM, or porcine NCCM-derived EVs or proteins. EV_UC_: NCCM-derived EVs obtained after size exclusion chromatography (SEC) and subsequent 100,000*g* ultracentrifugation (UC), P_UC_: NCCM-derived proteins obtained after SEC and subsequent 100,000*g* UC. Grey line represents controls (set at 100%). Black bars indicate significant differences between conditions (*p*<0.05); *, **,***: significantly different from controls (*p*<0.05, *p*<0.01, *p*<0.001, respectively). *n* = 6 porcine NCCM, EV, and P donors tested on a pool of 4 canine CLC donors. GAG: glycosaminoglycan. n.s.: not significantly different.

**Figure 6:**
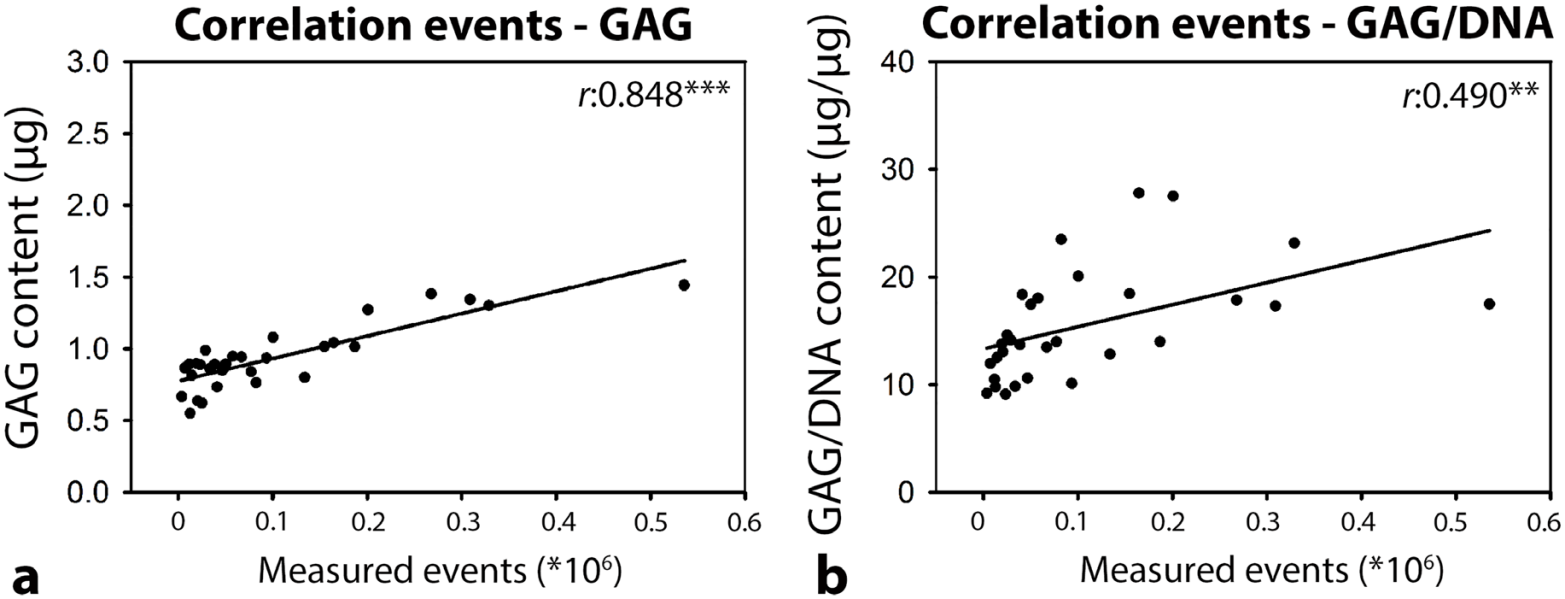
Pearson correlation between the total number of EVs (as determined by high-resolution flow cytometry) in the sucrose fractions of the porcine notochordal cell-conditioned medium (NCCM) extracellular vesicle (EV) pellets after size exclusion chromatography (SEC) and 100,000*g* ultracentrifugation (UC) and the GAG **(a)** and GAG/DNA **(b)** content of canine CLC micro-aggregates treated with a serial dilution of EV_UC_ (NCCM-derived EVs obtained after SEC and subsequent 100,000*g* UC). *n* = 6 porcine NCCM donors tested on a pool of 4 canine CLC donors. **,***: significant correlation (*p*<0.01, *p*<0.001, respectively). GAG: glycosaminoglycan.

In follow-up experiments, canine CLC micro-aggregates were cultured for 7 days in serial (1-16 times) dilutions of unfractionated NCCM, EV_UC_, and P_UC_ to determine the dose-dependency. A considerable amount of GAGs was present in P_qEV_ and especially EV_qEV_, whereas this was almost absent in EV_UC_ and P_UC_ media. Therefore, P_UC_ and EV_UC_ were used in this experiment to exclude GAG interference. No treatment significantly influenced the micro-aggregates’ DNA content (Figure 5a). The micro-aggregates’ GAG content was significantly induced by one until sixteen times diluted unfractionated NCCM (*p*<0.05, Figure 5b). NCCM-derived EVs and proteins also induced the micro-aggregates’ GAG content, but only until a concentration as present in two (EV_UC_) or four (P_UC_) times diluted NCCM (*p*<0.05). The micro-aggregates’ GAG content significantly decreased with serial dilution of unfractionated NCCM, EV_UC,_ and P_UC_. The micro-aggregates’ GAG/DNA content was only significantly induced by one times concentrated unfractionated NCCM, NCCM-derived EVs and proteins (*p*<0.05, Figure 5c). The micro-aggregates’ GAG/DNA content significantly decreased with serial dilution of NCCM-derived EVs, and unfractionated NCCM, but not with serial dilution of NCCM-derived proteins. There was a significant correlation between the total number of EVs in the porcine NCCM 100,000*g* EV UC pellets and the GAG (*r*:0.848, *p*<0.001) and GAG/DNA (*r*:0.490, *p*<0.01) content of the canine micro-aggregates treated with these EVs (Figure 6).

## DISCUSSION

### Porcine NCCM contained a considerable amount of EVs which exerted regenerative effects on CLCs

Using SEC, differential UC, density gradient floatation, and quantitative high-resolution flow cytometric analysis, this study is the first to demonstrate that porcine NCs secrete a considerable amount of EVs. We furthermore demonstrate that these EVs have biologic effects across species that suffer from clinical intervertebral disc disease. Notably, NCCM-derived EVs induced GAG deposition in canine CLCs to a comparable level as NCCM-derived soluble proteins and unfractionated NCCM. In contrast, NCCM-derived EVs increased the DNA and GAG content of human CLC micro-aggregates to a similar level as NCCM-derived proteins, but to a lesser extent than unfractionated NCCM. These species-dependent differences could be explained by differences in culture period. Canine micro-aggregate disintegration (in NCCM-derived media, but not in basal culture medium) prohibited a 21-day culture. As such, the 7-day canine CLC culture may be too short to demonstrate the full regenerative potential of NCCM. With serial dilution, NCCM-derived EVs and proteins lost their regenerative effect on canine CLCs earlier than unfractionated NCCM. Taken together, this may indicate that also in canine CLCs, unfractionated NCCM may have a stronger regenerative potential than NCCM-derived EVs or proteins employed at the same concentration. Based on their pronounced loss of biological activity with serial dilution compared with unfractionated NCCM, the EVs may interact with proteins present in NCCM. Notably, there were still some EVs present in the P_UC_ pellets, which are probably smaller in size given the SEC procedure. We cannot exclude that these EVs are another, possibly very potent EV subset. They may, however, also be part of the size continuum of the functional EVs (*e.g*. not another type, but only smaller EVs).

### Correlation between the number of EVs and their regenerative effect

In human CLCs, no linear correlation was found between the number of EVs (when applied at a concentration as present in undiluted NCCM) and their regenerative effect. Previous work could also not find an NCCM dose-dependent effect for neuronal inhibition [20]. In canine CLCs, however, we found a moderate correlation between the number of EVs and the micro-aggregates’ GAG/DNA content after 7-day EV_qEV_ treatment. These results can possibly be explained by the difference in culture period between the two species. After 7 days, canine CLCs were presumably in a dynamic phase of ECM production. After the 21-day human culture, however, secreted GAGs have already been deposited and only show a cumulative effect over time. Additionally, with serial dilution we found a significant positive correlation between the number of EVs and the GAG content of the canine micro-aggregates treated with these EVs. In conclusion, the results of this study may indicate that EVs induce a dose-dependent regenerative effect. However, a maximal response may be reached at an EV concentration as present in undiluted NCCM and/or after 21-day treatment. It remains to be determined if the EV-mediated anabolic effect can be further maximized with higher EV numbers.

### Ultracentrifugation mildly affected the biological activity of EVs on human, but not on canine CLCs

In the current study, the effect of 100,000*g* UC was compared with SEC-based EV purification, since previous work indicated that UC may not fully preserve the EV biological properties [27, 31]. In canine CLCs, 100,000*g* UC did not affect the biological activity of NC-derived EVs. In contrast, in human CLCs, UC slightly reduced the biological effect of EVs, since the DNA and GAG content of EV_qEV_-treated micro-aggregates was slightly higher than those of EV_UC_-treated micro-aggregates. Altogether, we have indications that 100,000*g* UC only mildly affected the biological activity of NCCM-derived EVs on CLCs from degenerated IVDs. Interestingly, collagen type I deposition was similarly induced by all treatments and collagen type II was only mildly deposited in EV_UC_- and P_UC_-treated micro-aggregates. To explain the slight differences in deposited ECM, a comprehensive analysis of the different EV preparations needs to be performed.

### NC-derived EVs exert regenerative effects in the absence of GAGs

When EVs were purified with SEC alone, interfering ECM residues (GAGs and collagens) were present. Affirmatively, previous work indicated that preparations obtained by limited processing resulted in ECM-embedded EVs, whereas sequential UC removed this contamination [28]. EVs bind to ECM compartments such as GAGs [32], collagens [33, 34], and hyaluronic acid [35] using integrins and other cell surface receptors such as CD44. With UC, interfering GAGs were lost from the preparations. Importantly, EV_UC_ and P_UC_ devoid of GAGs induced clear regenerative effects, indicating that the regenerative effects of NCCM-derived media were not mediated by GAGs. This finding seems to be in contrast with the notion that the NC-secreted GAG chondroitin sulphate is responsible for NCCM-induced neurite [20] and angiogenesis [36] inhibition. However, it cannot be excluded that neuro- and angiogenesis are differently regulated processes than ECM production.

ECM compartments may provide a favorable micro-environment for CLCs [37]. Unlike GAGs, collagens were not lost with UC. For this reason, we could not determine whether NC-secreted EVs/proteins or collagens were responsible for the regenerative effect induced by EV_UC_ and P_UC_. Collagen type I or II are the most abundant collagen types in the AF and NP, respectively [12, 38]. When collagen type I and II were supplemented at 0.5 mg/mL (a concentration at which collagen is present in NCCM), they did not induce regenerative effects in CLCs. This may suggest that the regenerative effects of EV_UC_ and P_UC_ were not caused by collagens, but by NC-secreted EVs and other proteins, respectively. Supplementation of 2.0 mg/mL collagen type II, but not collagen type I, induced GAG deposition in canine CLCs. This is in accordance with previous work on human MSCs, in which collagen type II activated MAPK/ERK signalling and was involved in Smad signalling [37, 39].

### Conclusions and future perspectives

In conclusion, porcine NCCM contained a considerable amount of EVs. These NC-derived EVs exerted comparable anabolic effects as NCCM-derived proteins, while unfractionated NCCM was more potent in human CLCs. The results of this study further imply that NCCM-derived GAGs and collagens were not responsible for the observed EV-mediated effects. Thus, NC-derived EVs have true regenerative potential. Based on their pronounced loss of biological activity with serial dilution compared with unfractionated NCCM, the EVs may interact with proteins present in NCCM. With a clinical directive in mind, the optimal combination of NC-secreted factors and the role of the EV as carrier need to be determined to fully exploit the regenerative potential of NC-derived treatment strategies. The next step would be to identify the bioactive substances which are transported with the EVs and the subsets of EVs that contain them. Thereafter, the (bioactive substances of the) EV subsets of interest could be used in functional *in vivo* studies.

## MATERIALS AND METHODS

### Sources of porcine NC-rich NP tissue and generation of NCCM

Healthy NP tissue was collected from 7 complete porcine spines (1.5 months of age, Thompson grade I) from the slaughterhouse in accordance with national regulations. To compare NCCM-derived EV and protein fractions with unfractionated NCCM in terms of EV and protein concentration, NCCM generation was slightly modified [12]. Briefly, NP tissue (1 gram/20 mL) was cultured for 4 days in hgDMEM+Glutamax (31966, Invitrogen) with 1% P/S (P11-010, GE Healthcare Life Sciences) at 37°C, 5% CO_2_, 5% O_2_. After 4 days, tissue was removed by 70 μm cell strainer filtration. The filtrate was centrifuged twice at 200*g* and 500*g* (ten minutes each, 4°C) to remove cells and to prevent the release of vesicles due to cell damage [40]. Subsequently, the supernatant was centrifuged at 4000*g* (45 minutes, 4°C) using 3 kDa Amicon Ultra-15 filter tubes (Merck Millipore) to remove small metabolites and waste products due to altered pH [20]. Substances with a molecular weight >3 kDa were suspended in fresh hgDMEM+Glutamax.

### Extracellular vesicle purification from porcine NCCM

EVs present within porcine NCCM were purified by differential UC [40] (Beckman Coulter Optima L-90K ultracentrifuge) and SEC. First, 37 mL of NCCM (previously centrifuged at 200, 500, and 4000*g*) was centrifuged at 10,000*g* (SW28 rotor; 4°C; 30 minutes; 8,700 rpm; RCF average 10,016*g*; RCF max 13,648*g*; κ-factor 2543.1) to remove cellular debris and apoptotic bodies. The supernatant was aliquoted and stored at -70°C until use. Per donor, 3 mL of 10,000*g* NCCM supernatant was subjected to qEV SEC-Columns (iZON Science; 1 mL/column) according to the manufacturer’s instructions (Figure 1). The qEV columns were calibrated and eluted with either sterile hgDMEM+Glutamax (one column per donor; control, EV_qEV_ and P_qEV_ conditions, see later) or PBS/0.1% BSA (two columns per donor; EV_UC_ and P_UC_ conditions, see later) before use. PBS/0.1% BSA was depleted from aggregates by overnight (O/N) UC at 100,000*g*. Twenty-five fractions of 0.5 mL were collected per qEV column and the respective protein concentration was determined (Nanodrop 2000, A280). Based on expected EV sizes and measured protein content, the three fractions with most EVs (between fraction 6 and 11) and proteins (between fraction 18 and 24) were separately collected and pooled per donor (Supplementary Figure 3), yielding 4.5 mL enriched with EVs and 4.5 mL enriched with proteins per donor. One and a half mL hgDMEM+Glutamax with NCCM-derived EVs or proteins were directly employed in culture experiments (EV_qEV_ and P_qEV_). The remaining 3 mL PBS/0.1% BSA with EVs or proteins was topped up with aggregate-depleted PBS/0.1% BSA in SW40 tubes and ultracentrifuged at 100,000*g* (SW40 rotor; 4°C; 65 minutes; 23000 rpm; RCF average 10,016*g*; RCF max 13,648*g*; κ-factor 2543.1). The 100,000*g* UC pellets of the pooled EV and protein fractions were separately resuspended in 40 μL aggregate-depleted PBS/0.1% BSA. Thirty-five μL of this 40 μL were employed in culture experiments (EV_UC_ and P_UC_). To the remaining 5 μL of the 100,000*g* UC pellets, 15 μL aggregate-depleted PBS/0.1% BSA was added, yielding 20 μL that where further employed for EV characterization.

### NCCM extracellular vesicle characterization

The EVs present in both 100,000*g* UC pellets (containing either EVs or proteins) were labelled with PKH67 (MIDI67, Sigma-Aldrich), floated to their buoyant density by O/N sucrose density gradient floatation, and quantitatively analyzed by high-resolution flow cytometry (BD Influx) as described previously [40]. In brief, 100,000*g* UC pellets were mixed with 180 μL Diluent C (MIDI67, Sigma-Aldrich) and 1.5 μL PKH67 and incubated for 3 minutes in SW40 tubes. One hundred μL IMDM (BE12-726F, Lonza) containing 10% EV-depleted Fetal Bovine Serum (generated by O/N UC at 100,000*g*) was added to stop the labelling process. These suspensions were mixed with 1.5 mL 2.5 M sucrose and overlaid with fifteen 700 μL sucrose fractions with decreasing molarity (2.0 until 0.4 M) in SW40 tubes. Sucrose gradients were then subjected to density gradient floatation for 16 hours at 200,000*g* (SW40 rotor; 4°C; 39,000 rpm; RCF average 192,072*g*; RCF max 270,519*g*; κ-factor 144.5). Twelve sucrose gradient fractions of 1 mL were collected from which the density was determined by refractometry. Lastly, density fractions were 20 times diluted in PBS (50 μL sample + 950 μL PBS) and analyzed for EV content using high-resolution flow cytometry, according to an earlier described method [40]. Detergents (0.1% Triton X-100 and 0.1% SDS) were added to the fraction with the highest number of events to determine whether measured events were truly EVs [29]. Additionally, serial dilution of the fraction with the highest number of measured events was performed to exclude swarm detection [30]. Data were analyzed using FlowJo software.

### Cell culture

Canine and human CLCs were collected from early degenerated IVDs (Thompson score II-III) as described previously [12]. Briefly, NP tissue was digested with 0.15% pronase (45 minutes) and 0.15% collagenase type II (O/N) at 37°C. Complete canine spines were collected from dogs euthanized in unrelated research studies, approved by the Utrecht University Animal Ethics Committee. Human IVDs (L2-L5, ≤48 hours after death) were obtained in the course of standard post mortem diagnostics, as approved by the scientific committee of the Pathology department of the University Medical Centre Utrecht (UMCU). Anonymous use of redundant tissue for research purposes is part of the standard treatment agreement with UMCU patients (Local Medical Ethical Committee number 12-364). The material was used in line with the code ‘Proper Secondary Use of Human Tissue’, installed by the Federation of Biomedical Scientific Societies.

CLCs from four canine (2-10 years of age, Beagles) and four human (50-63 years of age) donors were expanded as described previously [12]. At passage 2, the CLCs were pooled per species to assess the effect of donor-specific (EVs/proteins from) porcine NCCM on a representative human and canine CLC population. For micro-aggregate formation, 35,000 CLCs were plated per well in low-adherence cell-repellent surface 96-well plates (650970, CELLSTAR® Greiner Bio-one) in 50 μL basal culture medium (hgDMEM+Glutamax with 1% P/S, 1% ITS+ premix (354352, Corning Life Sciences), 0.04 mg/mL L-proline (P5607, Sigma-Aldrich), 0.1 mM Ascorbic acid 2-phosphate (A8960, Sigma-Aldrich), 1.25 mg/mL Bovine Serum Albumin (A9418, Sigma-Aldrich)) supplemented with 10 ng/mL TGF-β_1_ (240-B-010, R&D Systems). The 96-well plates were centrifuged at 50*g* for 5 minutes to induce micro-aggregate formation. The next day, the culture medium was replaced with (a) hgDMEM+Glutamax that underwent SEC using qEV columns (similarly as 10,000*g* NCCM supernatant; control), (b) 10,000*g* NCCM supernatant (NCCM), (c) NCCM-derived EVs obtained by SEC (EV_qEV_), (d) NCCM-derived proteins obtained by SEC (P_qEV_), (e) NCCM-derived EVs obtained by SEC followed by 100,000*g* UC (EV_UC_), or (f) NCCM-derived proteins obtained by SEC followed by 100,000*g* UC (P_UC_). All different culture media were supplemented with the factors as present in basal culture medium. EVs and proteins were applied to the CLCs at a similar concentration as present in 10,000*g* NCCM supernatant. EV_UC_ and P_UC_ conditions were included to determine whether 100,000*g* UC affected the biological activity of the NCCM-derived EVs/proteins. The micro-aggregates were cultured for 7 (canine) or 21 (human) days at 37°C, 5% CO_2_, 5% O_2_. Canine CLC micro-aggregates disintegrated after 7 days of culture in NCCM(-derived factors), which prevented a 21-day culture. Culture medium was changed twice weekly.

The micro-aggregates’ DNA (dsDNA High Sensitivity Assay Kit, Q32851, Invitrogen) and GAG content (DMMB assay [41]) were determined at day 7 (canine) or 21 (human) as described previously [12] (*n*=7, *in duplo* (human) or *triplo* (canine)). Since canine CLC micro-aggregates disintegrated after about 7 days of culture in NCCM(-derived factors), only human CLC micro-aggregates were histologically analyzed. Safranin O/Fast Green staining and collagen type I and II immunohistochemistry were performed as described previously [12]. The collagen and GAG concentration of the different culture media was analyzed using a hydroxyproline [42] and DMMB [41] assay, respectively.

In follow up culture experiments, canine CLCs were cultured for 7 days in basal culture medium, supplemented with or without 0.5, 1 and 2 mg/mL collagen type I (C9791, Sigma-Aldrich) or II (C9301, Sigma-Aldrich) to determine whether collagen (applied at a concentration as present in NCCM, EV_qEV_, EV_UC_, P_qEV_ and P_UC_) exerted regenerative effects on CLCs. Lastly, canine CLC micro-aggregates were cultured for 7 days in serial dilutions of 10,000*g* NCCM supernatant (NCCM), EV_UC_, and P_UC_ (1, 1:2, 1:4, 1:8 and 1:16 times concentrated) to determine when NCCM(-derived factors) lost their regenerative potential. The different media were diluted in basal culture medium. The same canine CLC and porcine NCCM donors were used as described earlier. The micro-aggregates’ DNA and GAG content were determined as described previously.

### Statistical analysis

Statistical analysis was performed using IBM SPSS (version 24). All data were examined for normal distribution (Shapiro Wilks test). Kruskal Wallis and Mann-Whitney U tests were performed on non-normally distributed data, whereas general linear regression models based on ANOVAs were used for normally distributed data. Benjamini & Hochberg False Discovery Rate *post-hoc* corrections for multiple comparisons were performed. To find correlations between the number of measured events and the micro-aggregates’ GAG(/DNA) content, Pearsons correlations were determined. A *p*-value <0.05 was considered significant.

## SUPPLEMENTARY MATERIALS FIGURES



## Abbreviations

AF: annulus fibrosus
CLC: chondrocyte-like cell
CTGF: connective tissue growth factor
ECM: extracellular matrix
EV: extracellular vesicles
GAG: glycosaminoglycan
IVD: intervertebral disc
MSC: mesenchymal stromal cell
NC: notochordal cell
NCCM: notochordal cell-conditioned medium
NP: nucleus pulposus
P: protein
SEC: size-exclusion chromatography
TGF-β_1_: transforming growth factor-β_1_
UC: ultracentrifugation
