# Supplementary material for: Notochordal-cell derived extracellular vesicles exert regenerative effects on canine and human nucleus pulposus cells
Source: Oncotarget. 2017 Oct 4;8(51):88845–56. doi: 10.18632/oncotarget.21483 (PMC5687651; doi:10.18632/oncotarget.21483)
Supplement: Supplementary file 1 [file oncotarget-08-88845-s001.pdf]

Notochordal-cell derived extracellular vesicles exert regenerative effects on canine and human nucleus pulposus cells

SUPPLEMENTARY MATERIALS

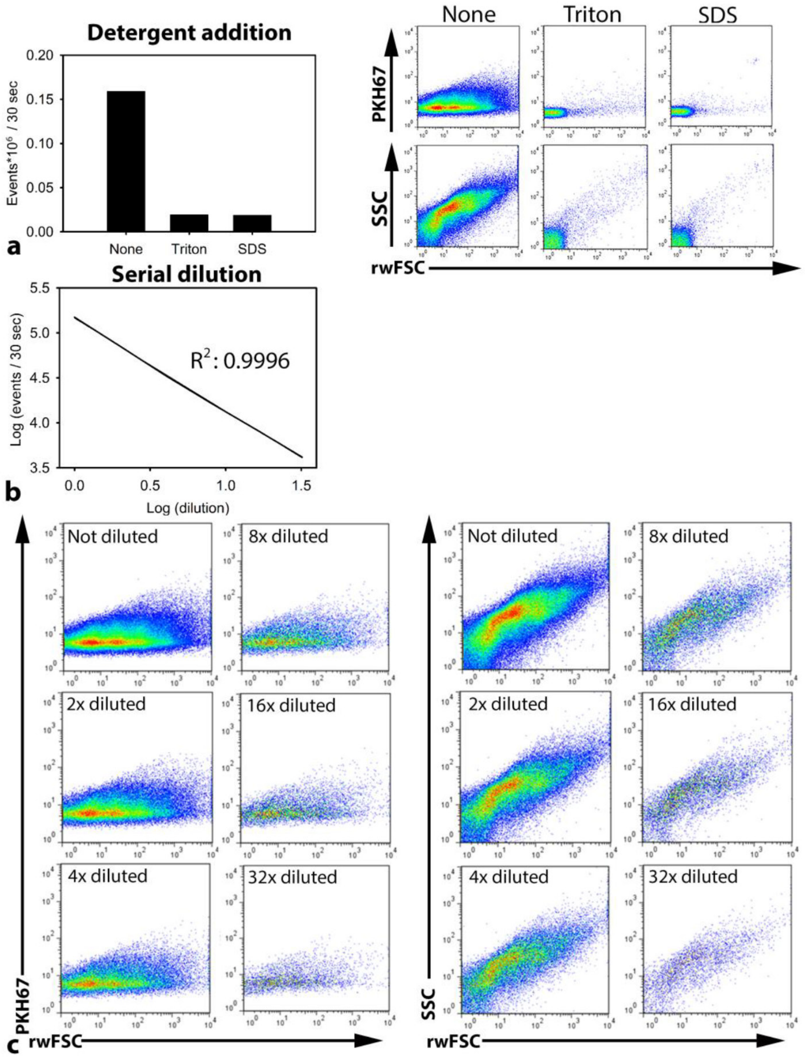

Supplementary Figure 1: Detergent addition to and serial dilution of EV sucrose gradient fraction. Detergent addition to the sucrose gradient fraction with the highest number of measured events from the porcine NCCM EV 100,000*g* ultracentrifugation pellet indicates that true EVs were detected with quantitative EV analysis (high resolution flow cytometry). Additionally, serial dilution of this fraction indicates that no swarm (coincident occurrence of multiple particles in the probe volume at the same time) was detected. Graphs and dot plots are depicted from one representative donor. **(a)** Addition of detergents (0.1% Triton or 0.1% SDS), that disrupt the lipid bilayer of the EVs, to the sucrose gradient fraction with the highest number of events (density 1.12 g/mL) reduced the event rate considerably, indicating that the measured events in this density fraction were indeed EVs. Moreover, light scattering events disappeared in the dot plots of the fraction treated with detergents. Dot plots represent levels of PKH67 intensity of side scatter (SSC) (y-axis) versus reduced wide-angle forward scatter (rwFSC) (x-axis). **(b)** To demonstrate the absence of swarm, a serial dilution on this sucrose gradient fraction (density 1.12 g/mL) was performed. A linear correlation (R^2: 0.9996) was detected between the number of measured events and the dilution of this sucrose gradient fraction, indicating that no swarm was detected. **(c)** The scatter pattern observed in the dot plots did not change with the serial dilution, further indicating that swarm was absent. Dot plots represent levels of PKH67 intensity (right) or side scatter (SCC; left) (y-axis) versus reduced wide-angle forward scatter (rw-FSC) (x-axis).

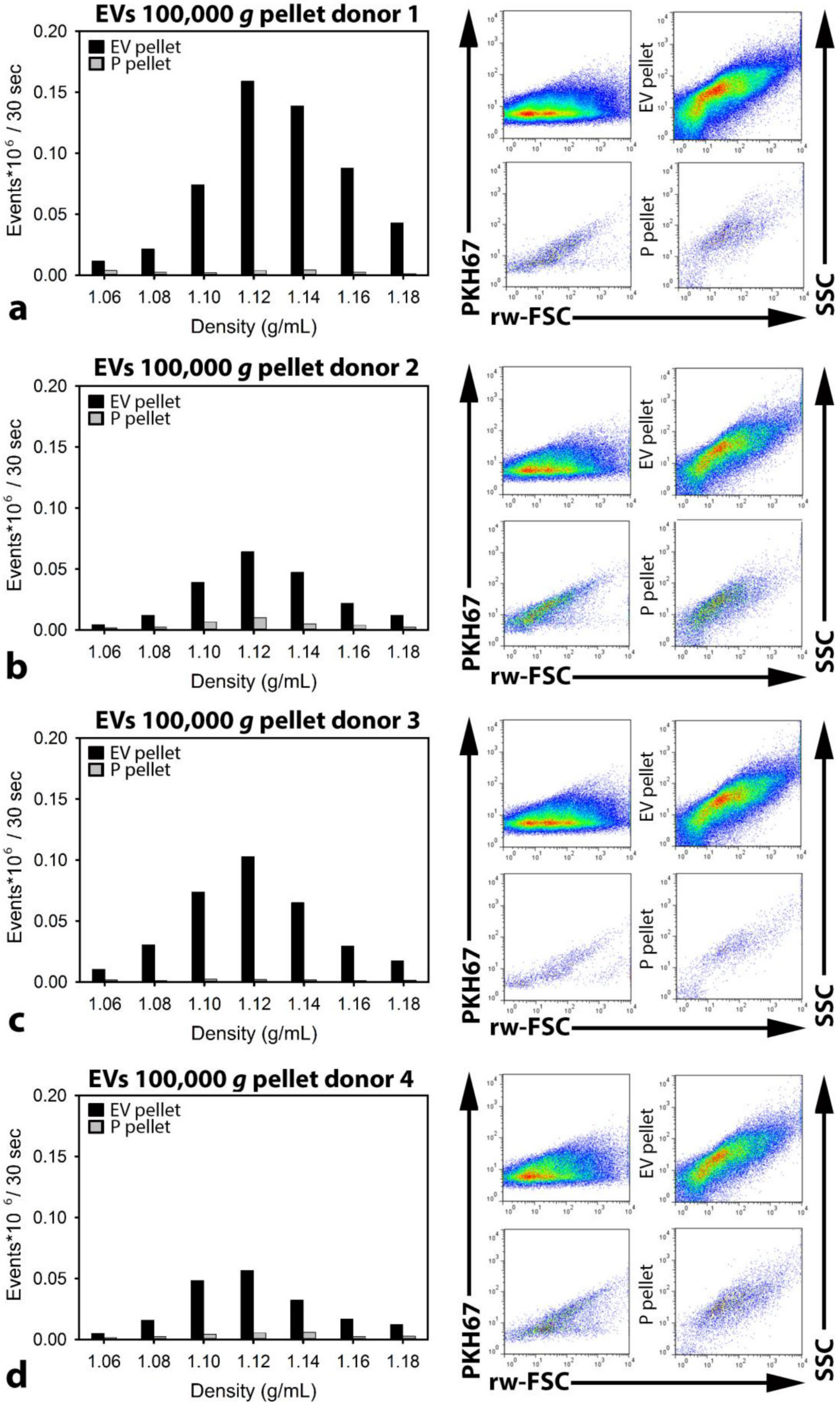
EVs 100,000 g pellet donor 1
EV pellet
P pellet
Events*10^6 / 30 sec
Density (g/mL)
a
PKH67
rw-FSC
SSC
EV pellet
P pellet
EVs 100,000 g pellet donor 2
b
EVs 100,000 g pellet donor 3
c
EVs 100,000 g pellet donor 4
d

(*Continued*)

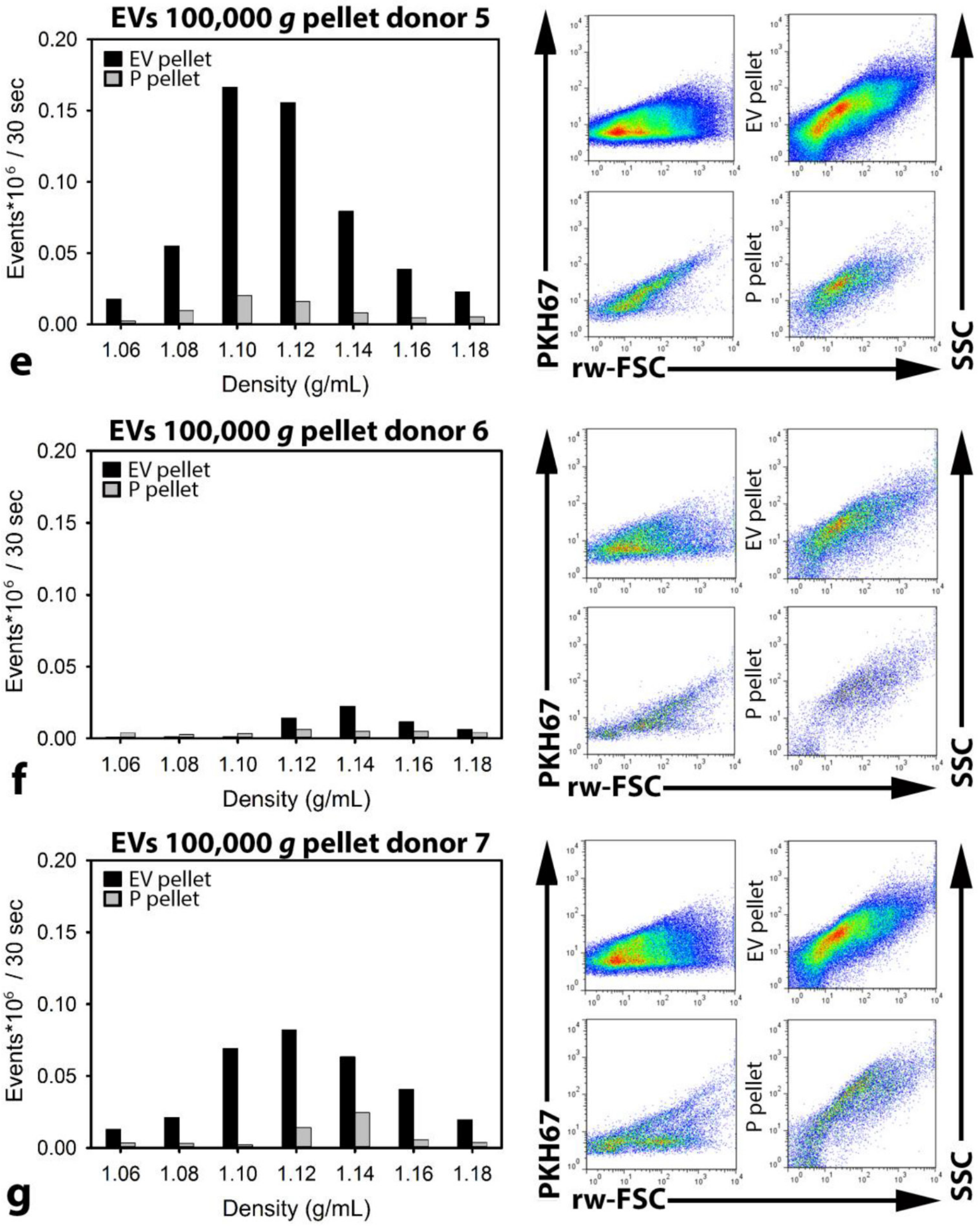

Supplementary Figure 2: Number of measured events and dot plots of porcine NCCM extracellular vesicle and protein 100,000g ultracentrifugation pellets. Quantitative flow cytometric extracellular vesicle (EV) analysis of the 100,000*g* ultracentrifugation pellets from all seven porcine notochordal cell-conditioned medium (NCCM) donors separately. High resolution flow cytometry of the porcine NCCM 100,000*g* ultracentrifugation pellets indicates that all donors demonstrate a similar pattern in number of measured events (EVs) per sucrose gradient fraction. Considerably more events were measured in the EV than in the protein (P) 100,000*g* pellet in all donors. In most donors, the highest number of measured events was present in the sucrose fraction with a density of 1.12 g/mL. Only the number of measured events per sucrose fraction differed considerably between donors. **(a-g)** Left: The number of measured events per sucrose gradient fraction (with different densities) from the porcine NCCM donors. Black bar: number of measured events per 30 sec in the sucrose gradient fractions of the EV 100,000*g* ultracentrifugation pellet. Grey bar: number of measured events per 30 sec in the sucrose gradient fractions of the P 100,000*g* ultracentrifugation pellet. Right: Dot plots of the sucrose gradient fraction with the highest number of measured events (density 1.12 g/mL) for most donors. Dot plots represent levels of PKH67 intensity (left) or side scatter (SCC; right) (y-axis) versus reduced wide-angle forward scatter (rw-FSC) (x-axis). Upper plots represent the 1.12 g/mL sucrose fraction of the EV 100,000*g* ultracentrifugation pellet, and lower plots represent the 1.12 g/mL sucrose fraction of the P 100,000*g* ultracentrifugation pellet.

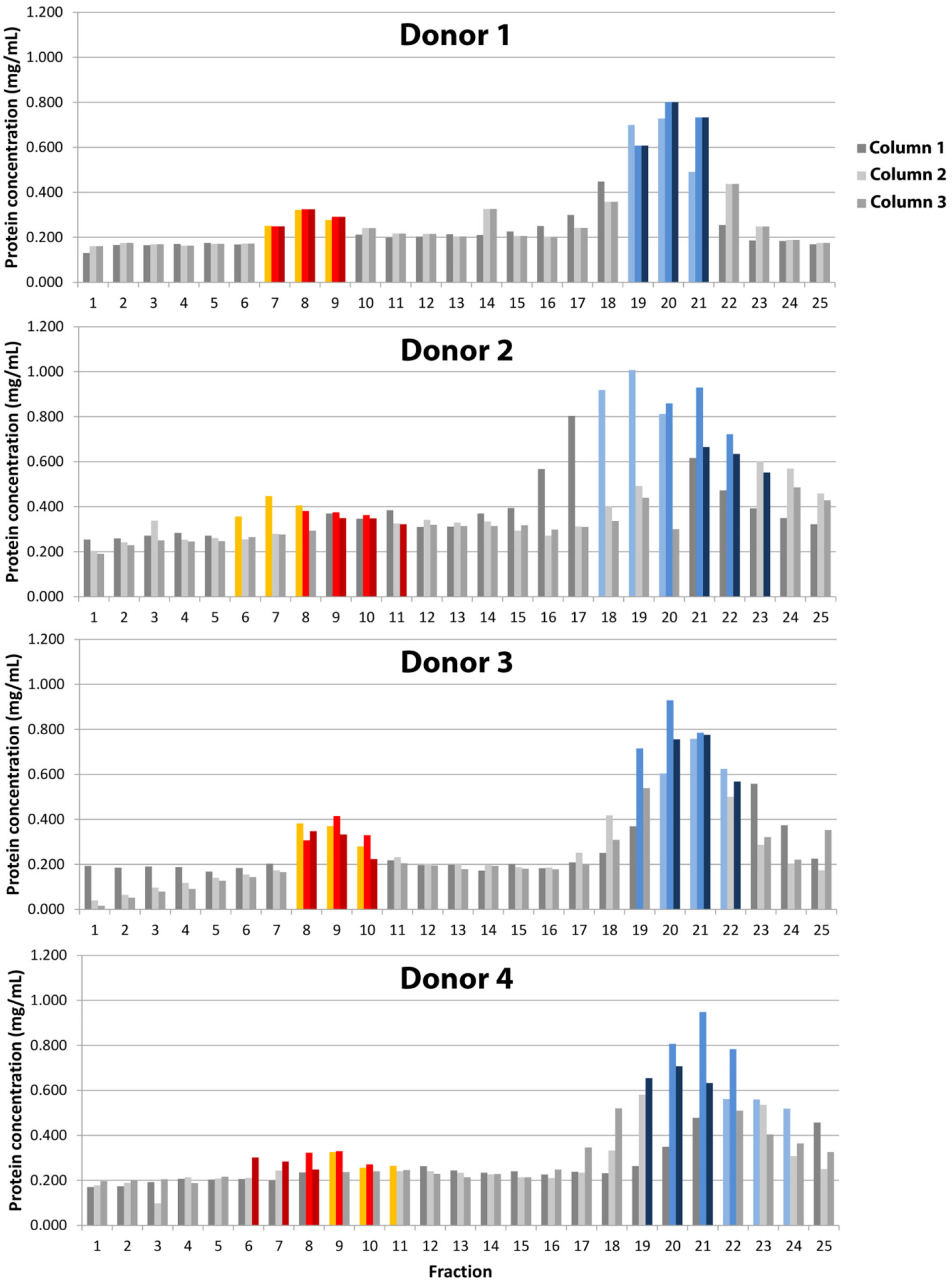
Donor 1
Donor 2
Donor 3
Donor 4
Protein concentration (mg/mL)
1.200
1.000
0.800
0.600
0.400
0.200
0.000
1 2 3 4 5 6 7 8 9 10 11 12 13 14 15 16 17 18 19 20 21 22 23 24 25
Fraction
Column 1
Column 2
Column 3

(*Continued*)

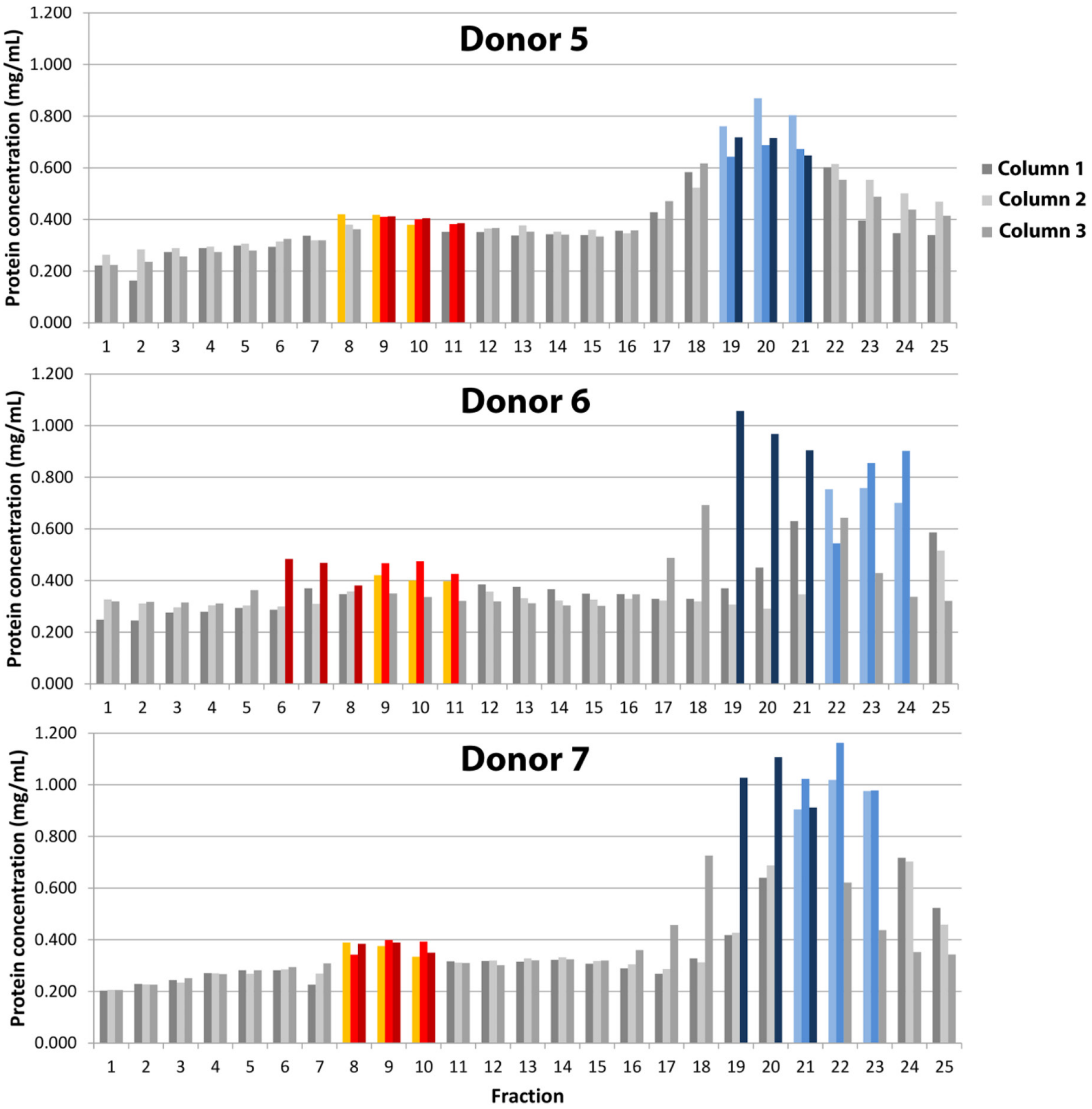

Supplementary Figure 3: Protein measurements of the different size exclusion colums fractions. Per porcine donor, 3 mL of 10,000*g* NCCM supernatant was subjected to qEV size exclusion columns (iZON Science; 1 mL/column). Three columns were used per donor (light-dark grey bars). Twenty-five fractions of 0.5 mL were collected per qEV size exclusion column and the respective protein concentration was determined (Nanodrop 2000, A280). Based on expected EV sizes and measured protein content, the three fractions with most EVs (between fraction 6 and 11; orange-red bars) and proteins (between fraction 18 and 24; light-dark blue bars) were separately collected and pooled per donor.

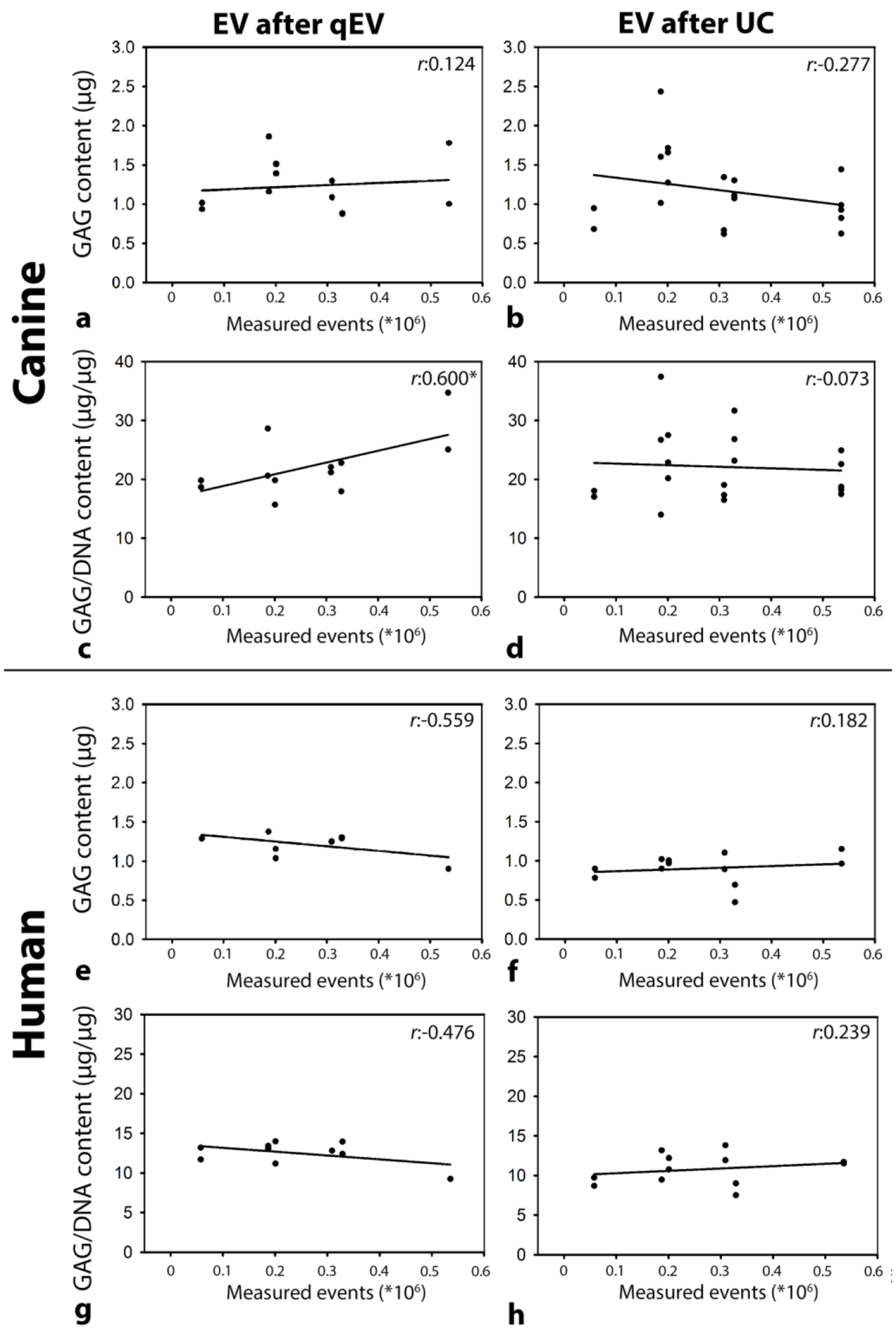

Supplementary Figure 4: Correlations between total number of measured events and the GAG content of canine and human CLC micro-aggregates treated with Evs. Pearson correlations between the total number of measured events in the sucrose fractions (1.06-1.18 g/mL) of the extracellular vesicle (EV) gradients and the GAG(/DNA) content of the canine and human chondrocyte-like cell (CLC) micro-aggregates treated with these EVs (after SEC (EV_{qEV}) and after SEC plus 100,000*g* ultracentrifugation (EV_{UC})). Correlation between the total number of measured events and the GAG content of the canine **(a)** and human **(e)** CLC micro-aggregates treated with EV_{qEV}, the GAG content of the canine **(b)** and human **(f)** CLC micro-aggregates treated with EV_{UC}, the GAG/DNA content of the canine **(c)** and human **(g)** CLC micro-aggregates treated with EV_{qEV}, and the GAG/DNA content of the canine **(d)** and human **(h)** CLC micro-aggregates treated with EV_{UC}. The results indicate that there is no significant correlation between the number of EVs and the GAG content of the canine and human CLC micro-aggregates treated with these EVs, except for the significant positive correlation between the number of events and the GAG/DNA content of canine micro-aggregates treated with EVs after qEV size exclusion. n = 6 porcine NCCM donors tested on a pool of 4 canine and 4 human CLC donors. *: significant correlation (p<0.05).
